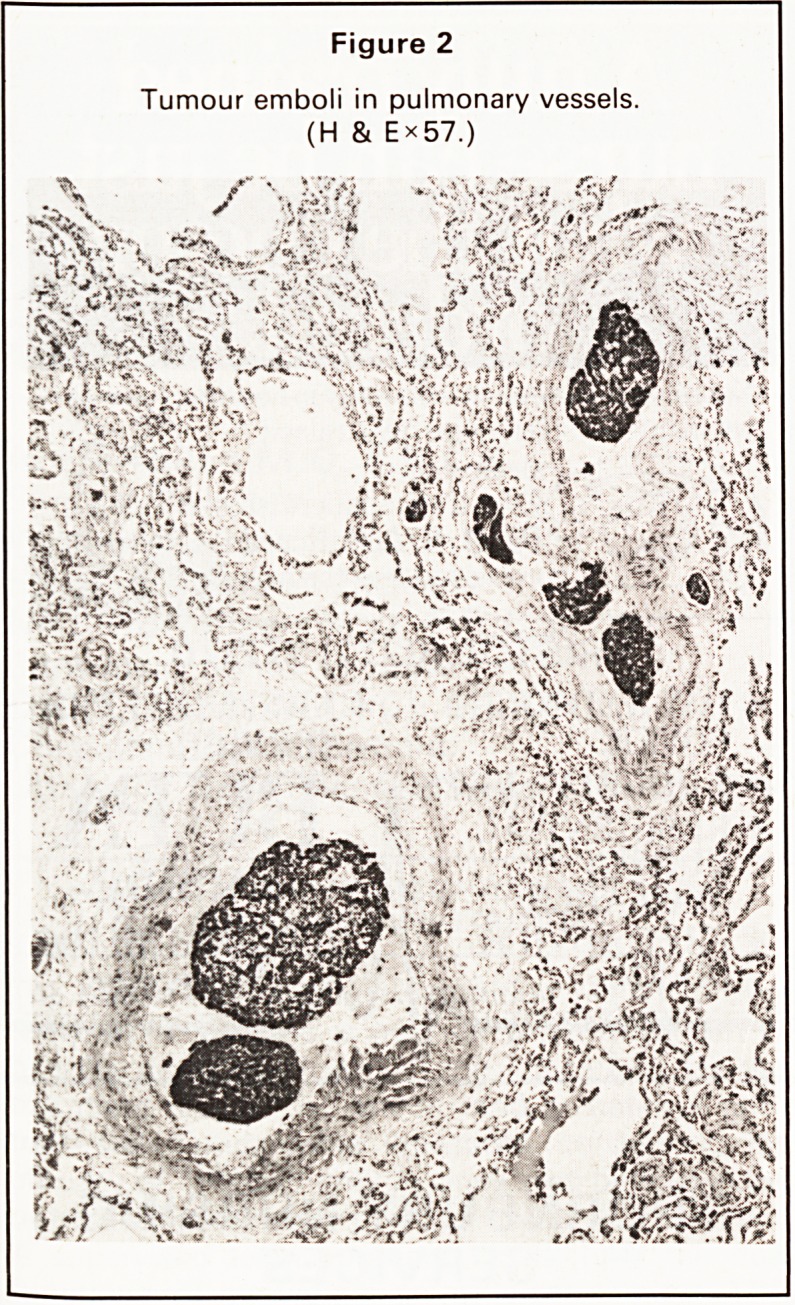# An Unusual Pulmonary Embolus

**Published:** 1984-01

**Authors:** John R. Fraser, M. Lansdown, Paul R. Goddard

**Affiliations:** Department of Radiodiagnosis; Pathology Department; Department of Radiodiagnosis Bristol Royal Infirmary


### An Unusual Pulmonary Embolus

**John R. Fraser**
Department of Radiodiagnosis
**M. Lansdown**
Pathology Department
**Paul R. Goddard**
Department of Radiodiagnosis
Bristol Royal Infirmary

### INTRODUCTION

The incidence of non-fatal pulmonary embolism in clinical practice has been estimated at 20 in 1000 hospital in-patients.[1] The origin of the emboli is usually assumed to be a site of venous thrombosis in the deep veins of the lower limb, or pelvis. We report a case of pulmonary embolism with an unusual embolic source.

### CASE HISTORY

Mrs. L., a 51 year old housewife, presented with a 2 cm. lump in the left breast, which she had first noticed three months previously. Excision biopsy revealed a very poorly differentiated adenocarcinoma with tumour in adjacent vessels. When radiotherapy was instituted 3 weeks later she complained of shortness of breath on exercise, and a dry cough. Over the following week she became increasingly dyspnoeic, tachypnoeic and cyanosed. Investigations included a chest radiograph, which was normal, and an isotope ventilation/perfusion scan, part of which is reproduced below (Figure 1). This shows multiple small perfusion defects in the right mid and upper zones without corresponding defects in ventilation, indicating pulmonary emboli. Anticoagulant therapy was started, but the patient deteriorated and died a week later.

**Figure 1**

Ventilation/perfusion scan. Anterior view. There are several areas of reduced activity in the right lung (arrows) on the perfusion scan, without a corresponding area of reduced ventilation.

### PATHOLOGICAL FINDINGS

At post mortem, the most striking feature was the presence of tumour deposits in pulmonary vessels, many of which could be defined as small arteries and arterioles (Figure 2). Some of these deposits were associated with thrombus formation. The lung parenchyma itself contained no tumour. Adenocarcinoma was also present in mediastinal lymph nodes and vertebral bone marrow, though there was no residual tumour in the breast. The necropsy diagnosis was carcinomatosis with multiple tumour emboli.

**Figure 2**

Tumour emboli in pulmonary vessels.
(H & E×57.)

### DISCUSSION

The value of isotope ventilation/perfusion scanning in the diagnosis of pulmonary thrombo-embolism is well established.[2] Other conditions may, however, produce 'mismatched' defects on the scan.[3] These include pulmonary vascular anomalies, pulmonary artery sarcoma, bronchogenic carcinoma and tumour embolism from a distant site, as in this case.

Tumour embolism presenting clinically with acute pulmonary embolism or pulmonary hypertension has been described in association with carcinoma of the liver,[4] breast,[5] kidney,[6] stomach,[7] colon and pancreas.[4] Other tumour sources reported include choriocarcinoma, neuroglastoma[8] and pelvic neoplasms.[9] In a review by the Department of Pathology at the University of Pittsburg, carcinoma of the stomach was the most frequent cause of tumour embolism to the lungs.[10] Another study of 1085 patients with solid malignant neoplasms showed that 24 (2.4%) had tumour emboli in the pulmonary arteries and arterioles, in the absence of significant parenchymal metastases.[11] Eight of these patients had suffered unexplained dyspnoea.

The tumour emboli in this case did not produce clinical effects until after the primary tumour had been removed. This is surprising since the primary tumour would, in most cases, be regarded as the source of the embolic material. It is possible, however, that the emboli were derived from deposits in the bone marrow.

The clinicopathological entity of tumour embolism to the pulmonary arteries must be distinguished from pulmonary thrombo-embolism which may occur in patients with venous thrombosis associated with malignant lesions.[12]

Approximately 3% of patients with thrombophlebitis have been found to have underlying malignancies.[13] Consideration of the possibility of tumour emboli in the differential diagnosis of pulmonary thrombo-embolism is more than academic. The tumours responsible for the emboli are mainly adenocarcinomas,[11] therapeutic regimens for which may be effective in reducing the frequency of embolism as the size of the primary tumour reduces.

In the many excellent review articles on Pulmonary Embolic Disease, which appear in the literature, tumour embolism is rarely mentioned. Our case emphasises this alternative embolic source in the clinical setting of pulmonary embolism.

### REFERENCES

1. SION, A. and LOPEZ-MAJANO V. (1978) Respiration **35**, 181.
2. VIAMANTE, M., KOOLPE, H., JANOWITZ, W. and HILDNER, F. (1980) Pulmonary thrombo-embolism—Update. *J.A.M.A.* 243 (21), 2229–34.
3. LI, D. K., SELTZER, S. E. and McNEIL, B. J. (1978) V/Q Mismatches Unassociated with Pulmonary Embolism: Case report and a Review of the Literature. *J.Nucl.Med.* 19 (12), 1331–1333.
4. BRISBANE, J. U., HOWELL, D. A. and BONKOWSKY, H. L. (1980) Pulmonary Hypertension as a Presentation of Hepatocarcinoma. Case History and a Review of the Literature. *Am.J.Med.* **68**, 466–469.
5. DURHAM, J. R., ASHLEY, P. F. and DORENCAMP, D. (1961) Cor Pulmonale due to Tumour Emboli. *J.A.M.A.* **175**, 107.
6. DAUGHTRY, J. C., STEWART B. H., GOLDING, L. A. R. and GROVES, L. K. (1977) Pulmonary Embolus presenting as the initial manifestation of renal cell carcinoma. *Ann.Thorac.Surg.* **24**, 178.
7. Case Report of the Massachussetts General Hospital (1980) (43) *N.E.J.M.* 303, (18), 1049–1056.
8. KEWW, W. S. et al: (1979) Eur. J. Paed. 132 (I), 61–6.
9. ARTHUS, D. S., STEPHENS, C. A., BRUMMIT, W. M., STEWARD, D. J. and NORMAN, M. G. (1973) Fatal tumour embolism during examination under anaesthesia. *Surgery* 74 (3), 466–468.

10. ALTEMUS, L. R. and LEE R. E. (1967) Carcinomatosis of the Lung with Pulmonary Hypertension: pathoradiologic spectrum. *Arch.Intern.Med.* **119**, 328.
11. KANE, R. D., HAWKINS, H. K., MILLER, J. A. and NOCE P. S. (1975) Microscopic pulmonary tumour emboli associated with dyspnoea. *Cancer* **36**, 1473–82.
12. LIEBERMAN, J. S., BORRERO, J., URDANETA, E. and WRIGHT J. S. (1961) Thrombophlebitis and Cancer. *J.A.M.A.* **177**, 542–545.
13. GREENBERG E., DIVERTIE M. B. and WOONER L. B. (1964) A review of unusual systemic manifestations associated with carcinoma. *Am.J.Med.* **36**, 106–120.

## Figures and Tables

**Figure 1 f1:**
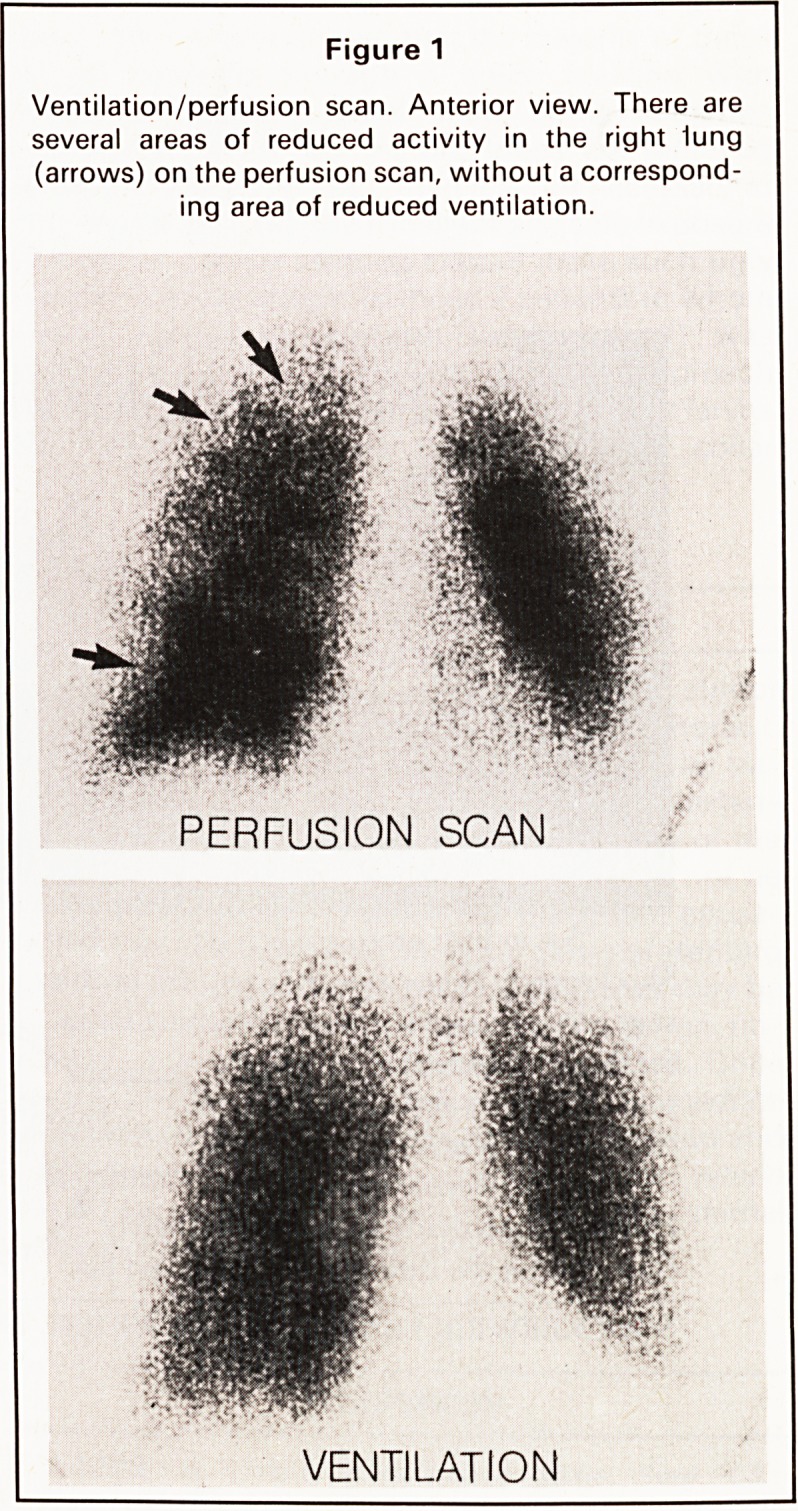


**Figure 2 f2:**